# Dietary vitamin B6 intake and gallstone prevalence in US adults: A cross-sectional analysis of NHANES 2017–2023

**DOI:** 10.1097/MD.0000000000050208

**Published:** 2026-08-14

**Authors:** Chengchen Liu, Nan Gao, Xun Yan, Ming Zhang, Ye Hong, Bo Hou, Kangkang Ji, Feng Lu

**Affiliations:** a Department of General Surgery, Binhai County People’s Hospital, Binhai Clinical College, Yangzhou University Medical College, Yancheng, China; b Department of General Surgery, Dongtai People’s Hospital, Dongtai, China; d Department of Respiratory and Critical Care Medicine, Binhai County People’s Hospital, Binhai Clinical College, Yangzhou University Medical College, Yancheng, China; c Department of Clinical Nutrition, Binhai County People’s Hospital, Yancheng, China

**Keywords:** Cross-sectional study, Dose-response, Gallstones, NHANES, Vitamin B6

## Abstract

Although mechanistic data implicate vitamin B6 in the regulation of cholesterol metabolism and inflammatory pathways, the epidemiological nexus between dietary vitamin B6 consumption and gallstone susceptibility remains largely uncharted in population-based cohorts. Leveraging cross-sectional data from the National Health and Nutrition Examination Survey (NHANES 2017–2023; n = 9273), we utilized weighted multivariable logistic regression to elucidate the association between vitamin B6 intake and self-reported gallstone history, with models rigorously adjusted for sociodemographic, lifestyle, and clinical confounders. Restricted cubic splines (RCS) were employed to delineate dose-response trajectories, while subgroup analyses served to identify potential effect modifiers. The overall prevalence of gallstones was 10.47% (971/9273). In fully adjusted models, a significant inverse correlation emerged between vitamin B6 intake and gallstone risk (OR per mg increase: 0.90; 95% CI: 0.82–0.99; *P* = .029). RCS analysis revealed a linear dose-response gradient, with statistical significance attained at intake levels exceeding 1.6615 mg/day (P for nonlinearity = 0.249). Stratified analyses indicated a modest yet robust inverse association among adults aged 20–39 years (OR: 0.75; 95% CI: 0.56–1.00), non-Hispanic Whites, individuals with a Poverty Income Ratio (PIR) > 4, those comorbid with diabetes or hypertension, and vigorous exercisers. Elevated dietary vitamin B6 intake correlates with a diminished prevalence of gallstones in the US adult population, particularly beyond a threshold of 1.66 mg/day. These findings suggest that specific metabolic and socioeconomic subgroups may derive disproportionate protective benefits, thereby offering critical insights for the formulation of targeted preventive interventions.

## 1. Introduction

Gallstone disease represents a prevalent health concern among American adults, with an estimated prevalence of approximately 10–15%.^[1,2]^ The underlying etiology involves complex interactions among imbalances in biliary cholesterol metabolism, gallbladder dysmotility, and chronic inflammation within the hepatobiliary microenvironment.^[3–7]^ Critically, these pathological processes contribute not only to symptomatic complications such as biliary colic and cholangitis but also to an elevated risk of hepatobiliary malignancies.^[8–10]^ While surgical intervention remains the primary therapeutic approach, identifying modifiable dietary protective factors holds significant promise for primary prevention strategies^[11,12]^。

Notably, vitamin B6 – a crucial coenzyme component – may influence stone pathogenesis through multiple metabolic pathways. Emerging evidence suggests its potential role in modulating homocysteine metabolism, facilitating reverse cholesterol transport, and mitigating oxidative stress, mechanisms relevant to renal calculi formation.^[13–16]^ Supporting this biochemical plausibility, a UK Biobank study revealed an inverse correlation between elevated plasma vitamin B6 levels and kidney stone prevalence.^[17]^ Furthermore, a recent investigation indicated that a high oxidative balance score, partially attributable to vitamin B6 intake, was associated with reduced gallstone risk.^[18]^ Although recent research has explored the role of dietary factors in gallstone risk, population-based studies have not yet comprehensively analyzed the independent association between dietary vitamin B6 intake and gallstone prevalence, underscoring the novel contribution of our investigation. This gap underscores the necessity for systematic assessments that account for population heterogeneity and methodological rigor to establish robust epidemiological evidence. Based on these considerations, we hypothesized that dietary vitamin B6 intake would exhibit a significant correlation with gallstone prevalence among US adults.

To address this hypothesis, we analyzed data from the National Health and Nutrition Examination Survey (NHANES 2017–2023). Our study design employed an integrated multidimensional analytical strategy to enhance precision: Implementation of weighted multivariable logistic regression models to ensure national representativeness and control for confounding; Stratification analyses to examine potential effect modification by key demographic and clinical factors; Comprehensive subgroup analyses to assess heterogeneity across socioeconomic status (income-to-poverty ratio, PIR), metabolic comorbidities (diabetes, hypertension), and physical activity levels. Additionally, restricted cubic spline (RCS) modeling was utilized to characterize potential nonlinear dose-response relationships. All models rigorously adjusted for covariates including sociodemographic factors, total energy intake, hydration status, and lifestyle variables.

The dual objectives of this investigation were: first, to elucidate the nuanced association between dietary vitamin B6 intake and gallstones through methodological optimization; and second, to establish an evidence-based framework for developing targeted nutritional interventions in high-risk metabolic subgroups.

## 2. Methods

### 2.1. Study population

**Figure 1.**
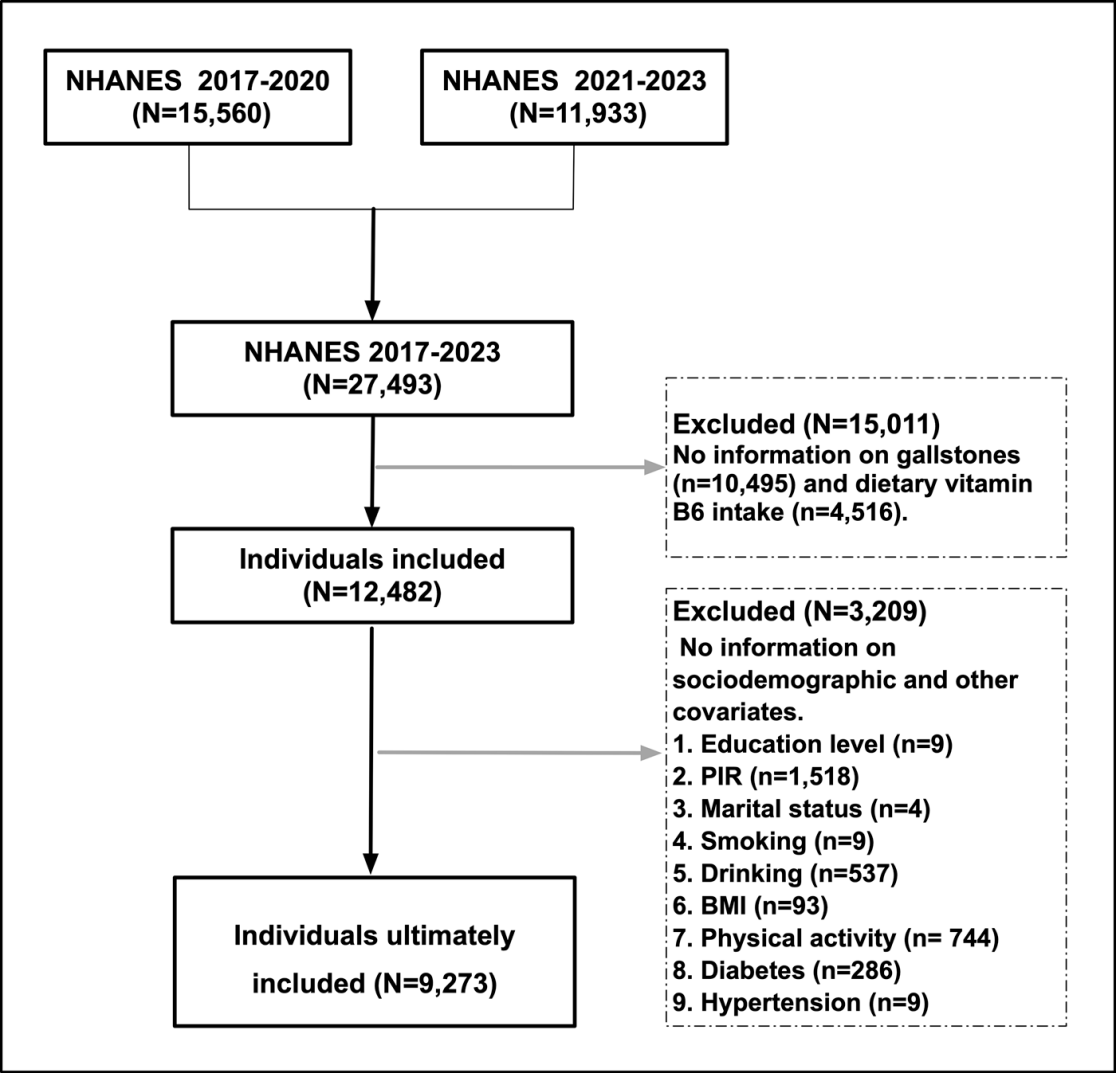
Study population selection flowchart. Participant inclusion and exclusion criteria for the NHANES 2017–2023 cohort. Gallstone status and dietary vitamin B6 data completeness determined eligibility. PIR = family income-to-poverty ratio, BMI = body mass index.

The National Health and Nutrition Examination Survey (NHANES) systematically collects nationally representative data on dietary status, health biomarkers, and chronic disease prevalence among noninstitutionalized U.S. residents through complex multistage sampling.^[19,20]^ This analysis utilized (2017–March 2020, 2021–2023). Inclusion required complete data on: gallstone status, dietary vitamin B6 intake, and key covariates. After applying exclusion criteria detailed in Figure 1, the final analytical sample comprised 9273 participants. All procedures adhered to STROBE guidelines for observational studies^[21]^ and NHANES ethical protocols. As this study involved the analysis of publicly available, de-identified data from the NHANES database, it was exempt from additional ethical review and did not require a separate institutional review board (IRB) approval by the authors’ institution.

### 2.2. Assessment of dietary vitamin B6 and gallstones

Vitamin B6 intake was quantified using the average of two 24-hour dietary recalls, weighted according to NHANES dietary day two sample weights to reflect national intake distributions.^[22]^ Gallstone diagnosis was ascertained through standardized medical questionnaire response to: “Has a doctor or other health professional ever told you that you had gallstones?.”^[23]^

### 2.3. Covariate classification

Covariates were selected a priori based on established gallstone risk factors^[1,23–25]^ and categorized as: Sociodemographics: Age (20–39, 40–59, ≥60 years), race/ethnicity (Hispanic, non-Hispanic White, non-Hispanic Black, other), education (< high school, high school, ≥college), marital status (NHANES-coded), and poverty-income ratio (PIR: <1.0, 1.0–4.0, >4.0).^[26]^ Lifestyle factors: Smoking status, alcohol consumption, and physical activity (sedentary, moderate, vigorous). Clinical metrics: Body mass index (BMI), diabetes (physician-diagnosed or medication use), hypertension (physician-diagnosed or antihypertensive use), total daily energy (kcal) and water intake (g).

### 2.4. Statistical analysis

Continuous variables are presented as medians with interquartile ranges (IQR), categorical variables as weighted counts and proportions. A tiered modeling approach employed weighted multivariable logistic regression to address sampling design and nonresponse bias: Model 1: Unadjusted; Model 2: Adjusted for sociodemographics (age, gender, race, education, PIR, marital status); Model 3: Fully adjusted (Model 2 + lifestyle + clinical covariates: smoking, alcohol, BMI, physical activity, diabetes, hypertension, energy intake, water intake). Association strength was expressed as odds ratios (OR) with 95% confidence intervals (95% CI). Subgroup analyses examined effect modification by age, sex, race, PIR, diabetes, hypertension, and physical activity. Dose-response relationships were characterized using restricted cubic splines (RCS) with 4 knots (5th, 35th, 65th, 95th percentiles), testing nonlinearity via likelihood ratio tests. Analyses were performed using SPSS 29.0, Stata/MP 18.0, and R 4.4.2. Statistical significance was defined as two-sided *P* < .05.

## 3. Results

### 3.1. Baseline description of participants

**Figure 2.**
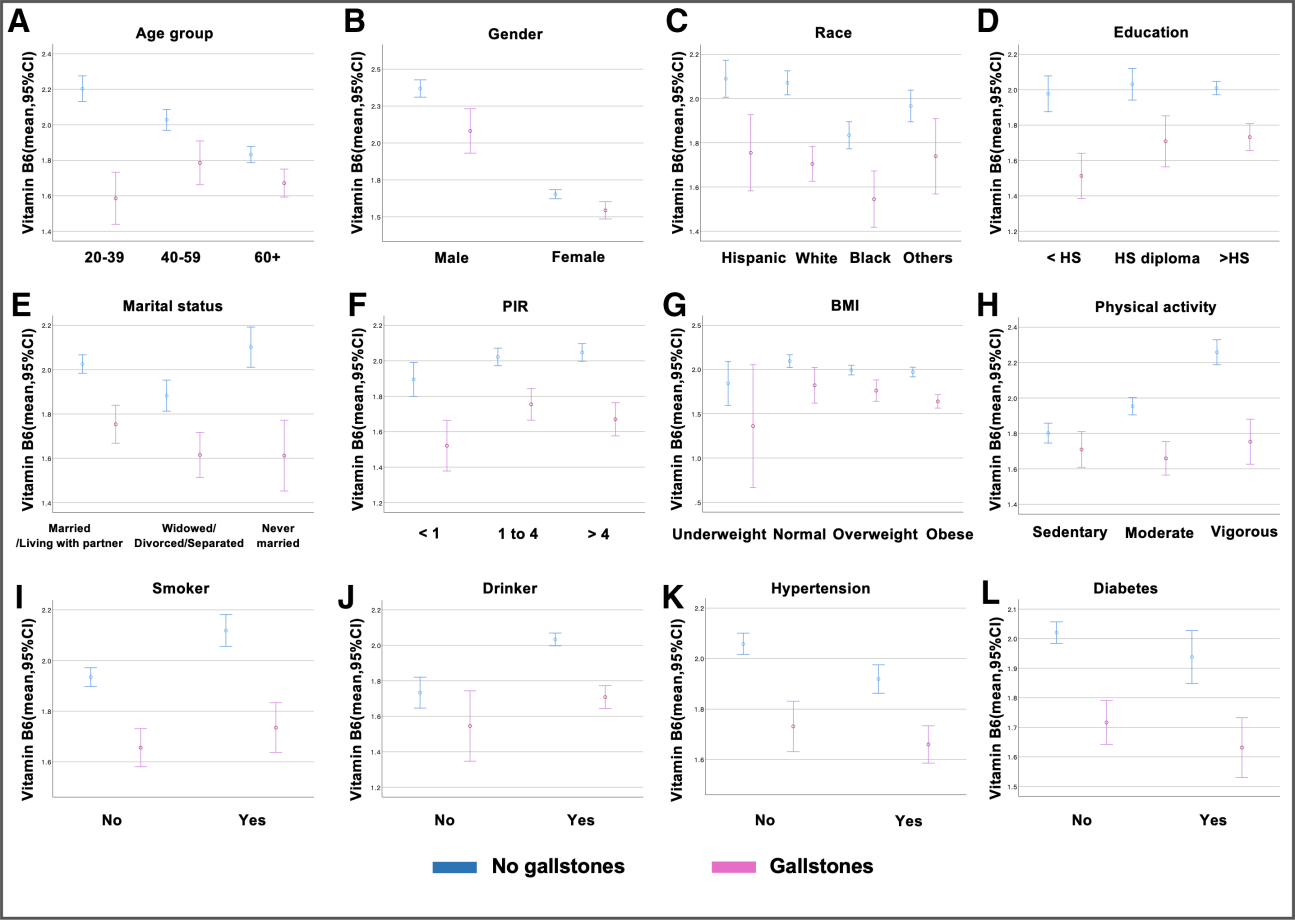
Stratified analysis of vitamin B6 intake by gallstone status and covariates. (A–L) Differences in dietary vitamin B6 intake (mean ± 95% CI) between adults with and without gallstones, stratified by sociodemographic and clinical factors. The inverse association was pronounced in younger, non-Hispanic White, low-education, and metabolically compromised subgroups.

Table 1 presents participant characteristics stratified by gallstone status. Among 9273 included individuals, gallstone prevalence was 10.47% (n = 971). Participants with gallstones exhibited significantly higher median age (*P* < .001) and BMI (*P* < .001), alongside lower vitamin B6 intake (*P* < .001), total energy consumption (*P* < .001), water intake (*P* = .036), and poverty-income ratios (PIR, *P* < .001) compared to gallstone-free individuals. Gallstone patients also demonstrated lower proportions of females (*P* < .001), smokers (*P* < .001), and sedentary individuals (*P* = .001), but higher rates of diabetes (*P* < .001) and hypertension (*P* < .001). Additionally, they had fewer college-educated (*P* = .011) and unmarried individuals (*P* < .001). Stratified analysis of vitamin B6 intake (Fig. 2) confirmed consistently lower consumption in gallstone patients across all subgroups (*P* < .001), with particularly pronounced deficits among young adults (20–39 years), males, Hispanic and non-Hispanic White individuals, those with less than high school education, never-married subjects, underweight individuals, vigorous exercisers, smokers, alcohol consumers, and hypertension-free participants.

**Table 1:** Baseline characteristics of study participants by gallstone status.

Characteristic	No gallstones N = 8302†	Gallstones N = 971†	*P*-value§
**Age (years**)‡	46 (32, 60)	59 (46, 69)	<.001
**BMI (kg/m^2^**)‡	28 (24, 33)	31 (27, 38)	<.001
**Vitamin B6 (mg**)‡	1.74 (1.22, 2.43)	1.52 (1.12, 2.07)	<.001
**Energy (kcal**)‡	1982 (1531, 2572)	1766 (1391, 2243)	<.001
**Water intake (g**)‡	2774 (2083, 3629)	2620 (2017, 3448)	.036
**PIR**	3.42 (1.77, 5.00)	2.92 (1.58, 4.80)	<.001
**Female, n (%**)	4168 (49%)	699 (74%)	<.001
**Race**			.026
Hispanic	1512 (15%)	205 (14%)	
Non-Hispanic White	3866 (65%)	503 (70%)	
Non-Hispanic Black	1758 (11%)	151 (6.8%)	
Others	1166 (9.5%)	112 (8.7%)	
**Education**			.011
<HS	1108 (8.4%)	142 (9.5%)	
HS diploma	1801 (25%)	234 (30%)	
College or above	5393 (67%)	595 (60%)	
**Marital status**			<.001
Married/Living with partner	4797 (63%)	557 (62%)	
Widowed/Divorced/Separated	1825 (17%)	291 (25%)	
Never married	1680 (20%)	123 (13%)	
**Smoker (current or former**)	3384 (40%)	470 (51%)	<.001
**Drinker (current or former**)	7641 (93.4%)	889 (93%)	<.001
**Physical activity**			.001
Sedentary	1935 (19%)	280 (25%)	
Moderate	3880 (46%)	477 (47%)	
Vigorous	2487 (35%)	214 (27%)	
**Diabetes, yes**	1087 (10%)	248 (21%)	<.001
**Hypertension, yes**	2905 (29%)	504 (48%)	<.001

† N not Missing (unweighted).

‡ Median (Q1, Q3); n (unweighted) (%).

§ Design-based Kruskal Wallis test; Pearson’s X^2: Rao & Scott adjustment. PIR, ratio of family income to poverty; BMI, body mass index.

### 3.2. Association between vitamin B6 intake and gallstones

**Figure 3.**
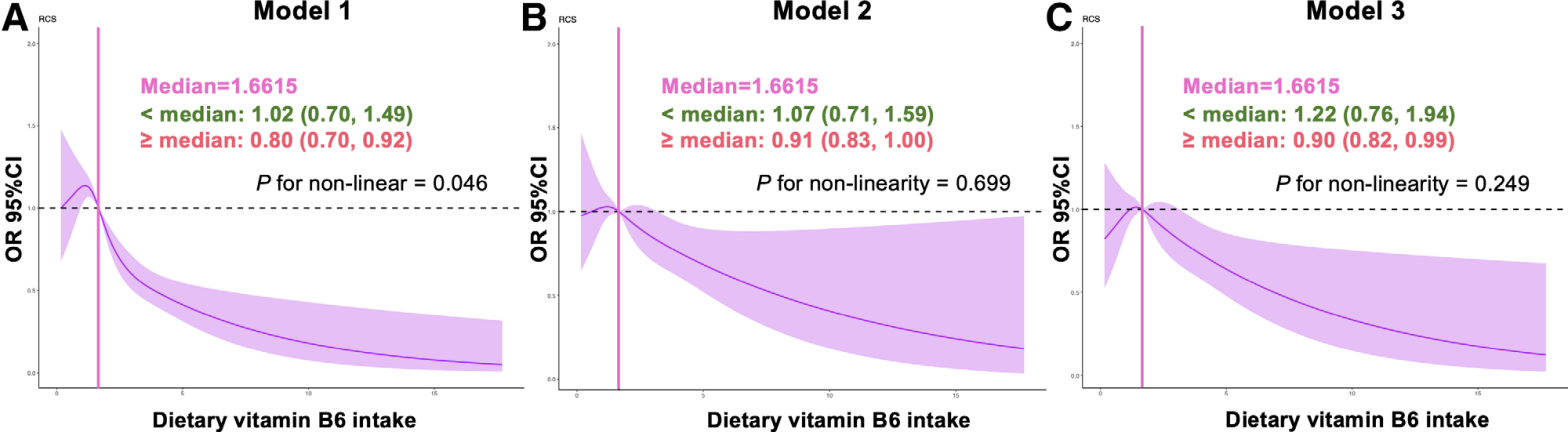
Dose-response relationship between vitamin B6 intake and gallstone risk. (A–C) Restricted cubic spline curves (knots at 10%, 50%, 90%) showing adjusted odds ratios (solid line) and 95% CIs (shaded area) for gallstones across vitamin B6 intake levels. Reference: median intake (1.66 mg). *P* for nonlinearity = 0.249 (Model 3).

Multivariable logistic regression identified vitamin B6 intake as a protective factor against gallstones (OR = 0.90, 95% CI:0.82–0.99; *P* = .029), while age (OR = 1.04, 95% CI:1.03–1.04; *P* < .001), BMI (OR = 1.06, 95% CI:1.05–1.08; *P* < .001), female sex (OR = 3.05, 95% CI:2.35–3.95; *P* < .001), and smoking (OR = 1.56, 95% CI:1.25–1.95; *P* < .001) emerged as risk factors (Table 2). Non-Hispanic Black ethnicity showed reduced risk (OR = 0.42, 95% CI:0.31–0.57; *P* < .001). In tiered regression models (Table 3), the inverse association remained significant in the fully adjusted Model 3 (per mg intake: OR = 0.90, 95% CI:0.82–0.99; per SD increase: OR = 0.86, 95%CI:0.74–0.98; *P* = .029). Restricted cubic spline analysis (Fig. 3) established a threshold effect: significant risk reduction occurred only above 1.665 mg/day vitamin B6 intake (*P* for nonlinear < 0.05).

**Table 2:** Multivariable logistic regression: associations between gallstones and predictors.

Variables	OR 95%CI	*P*-value
**Vitamin B6 (mg**)	0.90 (0.82, 0.99)	**.029**
**Age, y**	1.04 (1.03, 1.04)	**<.001**
**BMI, kg/m^2^**	1.06 (1.05, 1.08)	**<.001**
**Energy (kcal**)	1.00 (1.00, 1.00)	.725
**Water intake (g**)	1.00 (1.00, 1.00)	.276
**Female vs Male (*ref.***)	3.05 (2.35, 3.95)	**<.001**
**Race**		
Hispanic	***reference***	
Non-Hispanic White	0.79 (0.61, 1.02)	.066
Non-Hispanic Black	0.42 (0.31, 0.57)	**<.001**
Others	0.90 (0.63, 1.29)	.571
**Education**		
< HS	***reference***	
HS diploma	1.20 (0.84, 1.72)	.311
College or above	1.07 (0.77, 1.48)	.693
**Marital status**		
Married/Living with partner	***reference***	
Widowed/Divorced/Separated	0.86 (0.66, 1.12)	.257
Never married	1.09 (0.79, 1.51)	.593
**PIR**		
< 1	***reference***	
1 to 4	1.05 (0.73, 1.51)	.790
> 4	0.85 (0.56, 1.28)	.434
**Smoker (yes vs no**)	1.56 (1.25, 1.95)	**<.001**
**Drinker (yes vs no**)	0.97 (0.64, 1.46)	.868
**Physical activity**		
Sedentary	***reference***	
Moderate	1.01 (0.78, 1.29)	.960
Vigorous	1.20 (0.88, 1.63)	.252
**Diabetes (yes vs no**)	1.28 (0.98, 1.67)	.066
**Hypertension (yes vs no**)	1.17 (0.93, 1.48)	.183

Note: Adjusted odds ratios (ORs) and 95% confidence intervals (CIs) from the fully weighted model (Model 3). Covariates: age, sex, race, education, PIR, marital status, smoking, alcohol, BMI, physical activity, diabetes, hypertension, energy/water intake.

**Table 3:** Association between dietary vitamin B6 intake and gallstones across adjustment models.

Dietary vitamin B6 intake	^a^ Model 1		^b^ Model 2		^c^ Model 3	
	OR (95% CI)	*P* value	OR (95% CI)	*P* value	OR (95% CI)	*P* value
**Vitamin B6 (mg**)	0.78 (0.70, 0.86)	**<.001**	0.91 (0.84, 0.99)	**.024**	0.90 (0.82, 0.99)	**.029**
**Per 1 SD (SD = 1. 53 mg**)	0.68 (0.58, 0.79)		0.86 (0.76, 0.98)		0.86 (0.74, 0.98)	

Note: Incremental adjustment: Model 1 (crude), Model 2 (sociodemographic), Model 3 (full). ORs expressed per mg and per 1-SD (1.53 mg) increase in vitamin B6.

### 3.3. Subgroup and interaction analyses

**Figure 4.**
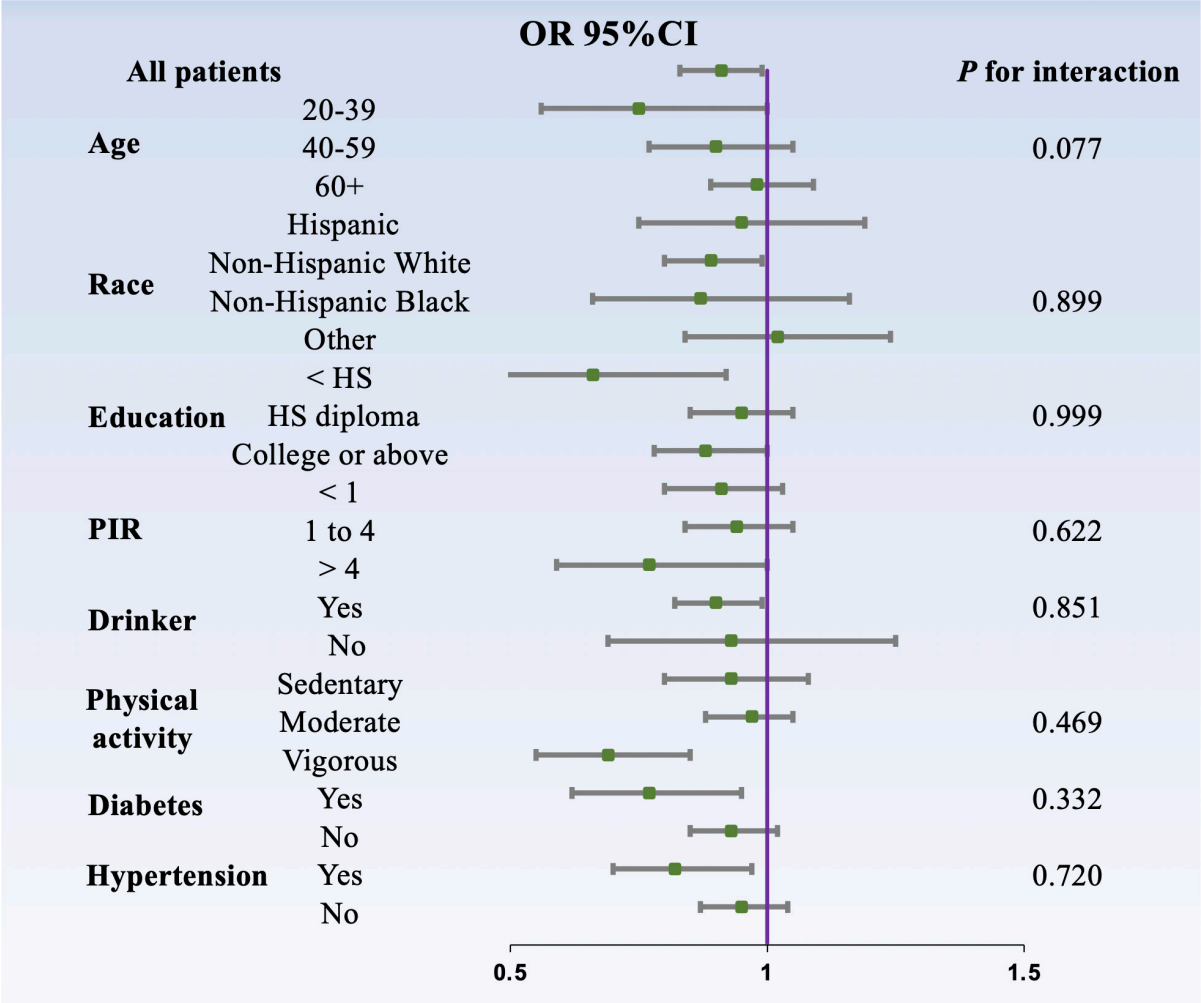
Subgroup analysis: sensitivity of vitamin B6–gallstone association to effect modifiers. Forest plot of adjusted ORs (95% CIs) for gallstones per mg increase in vitamin B6 intake, stratified by covariates. Interaction *P* values test effect modification. PIR = family income-to-poverty ratio, HS = high school.

Stratified analyses (Figure 4, Table S1, Supplemental Digital Content 1) revealed enhanced protective effects in specific subgroups: younger adults (20–39y: OR = 0.75, 95% CI:0.56–1.00), high-income individuals (PIR > 4: OR = 0.77, 95% CI:0.59–1.00), diabetics (OR = 0.77, 95% CI:0.62–0.95), vigorous exercisers (OR = 0.69, 95% CI:0.55–0.85), hypertensives (OR = 0.82, 95% CI:0.70–0.97), alcohol consumers (OR = 0.90, 95% CI:0.82–0.99), and those with low education (<HS: OR = 0.66, 95% CI:0.47–0.92). Formal interaction testing found no statistically significant effect modification by any covariate (all *P* for interaction > 0.05), indicating consistent directionality of the vitamin B6-gallstone association across population strata.

## 4. Discussion

This cross-sectional analysis of NHANES 2017–2023 data establishes for the first time a significant inverse association between dietary vitamin B6 intake and gallstone prevalence among US adults (OR per mg increase = 0.90; 95% CI: 0.82–0.99). Crucially, this association attained statistical significance at intakes exceeding 1.66 mg/day, thereby providing empirical validation for our initial hypothesis and addressing a critical knowledge gap regarding the independent relationship between dietary vitamin B6 and gallstone risk.

These epidemiological observations align robustly with emerging mechanistic evidence. Notably, the active form vitamin B6 (pyridoxal 5’-phosphate, PLP) modulates cholesterol homeostasis through multifaceted pathways. Experimental studies demonstrate PLP’s pivotal role in attenuating inflammation by regulating inflammatory vesicle activity, particularly through NLRP3 inflammasome suppression^[27]^ – a mechanism highly relevant to gallstone pathogenesis given that cholesterol crystals activate NLRP3, triggering mucin hypersecretion and stone nucleation.^[28]^ Furthermore, in models of drug-induced liver injury, vitamin B6 derivatives like pyridoxal isonicotinoyl hydrazone (PIH) promote cholesterol conversion to bile acids via CYP7A1 upregulation.^[29]^ Collectively, these biological mechanisms substantiate our population-level findings.

The clinically significant heterogeneity observed across subgroups merits emphasis. The pronounced numerically stronger associations among younger adults (20–39 years; OR = 0.75) likely reflects age-related differences in gallbladder motility and cholesterol saturation.^[30,31]^ Similarly, the suggestive patterns in diabetic patients (OR = 0.77) may arise from vitamin B6’s documented capacity to ameliorate insulin resistance.^[32–34]^ Of socioeconomic relevance, the enhanced benefit in high-income individuals (PIR > 4; OR = 0.77) potentially reflects superior dietary quality, including greater consumption of vitamin B6-rich sources like deep-sea fish and whole grains.^[35,36]^ However, all interaction *P*-values were > 0.05, indicating no statistically significant effect modification by these covariates. These stratified insights advance the paradigm for precision nutrition in gallstone prevention.

Importantly, our identified effective threshold (1.66 mg/day) closely aligns with the WHO’s recommended daily intake for adults (1.3–1.7 mg),^[37]^ rendering this a clinically feasible target. The threshold of ~1.66 mg/day should be interpreted cautiously, as the wide confidence intervals at higher intake levels (Fig. 3) indicate reduced precision in this range, likely due to fewer observations. Therefore, this estimate serves as a suggestive reference rather than a definitive cutoff. This dose-response relationship implies that protective effects manifest only beyond specific physiological thresholds. We thus propose prioritizing this intake in high-risk populations (e.g., metabolic syndrome, obesity) through dietary sources (chickpeas, tuna) or supplementation. Future randomized controlled trials (RCTs) should validate this approach while investigating potential synergies with other B vitamins.

Methodological strengths and limitations warrant careful consideration. Key strengths include rigorous adjustment for total energy intake, hydration status, and metabolic comorbidities via weighted multivariable modeling, enhancing association accuracy. Nevertheless, 3 limitations merit acknowledgment: First, the cross-sectional design precludes causal inference and cannot exclude reverse causation (e.g., gallstones altering dietary habits). Second, reliance on 24-hour dietary recalls may misrepresent habitual vitamin B6 intake. Third, residual confounding from unmeasured factors remains possible. Additionally, important dietary factors such as fiber intake, fatty acid composition, and caffeine consumption were not included due to data availability, and this could introduce residual confounding. Finally, the cross-sectional design and dietary assessment method further limit the certainty of this threshold. To translate these insights into clinical practice, future research should prioritize: prospective cohort studies to verify causality and address residual confounding; randomized controlled trials evaluating vitamin B6 supplementation efficacy in high-risk metabolic subgroups; and mechanistic investigations exploring nutrient synergies between B6 and related micronutrients. Such evidence will be essential for developing precise, nutrition-based primary prevention strategies against gallstone disease.

## 5. Conclusions

This cross-sectional investigation identified a modest inverse association... a statistically significant reduction in gallstone prevalence was observed at intakes above approximately 1.66 mg/day. This threshold should be interpreted cautiously and is considered suggestive pending prospective validation. Exploratory subgroup analyses suggested numerically stronger inverse associations among younger adults (20–39 years), individuals with metabolic comorbidities (diabetes/hypertension), and high socioeconomic status groups (PIR > 4), although interaction tests were not statistically significant. These hypothesis-generating observations require confirmation in prospective studies. These findings collectively advance our understanding of vitamin B6 as a modifiable protective factor against gallstone formation through cholesterol homeostasis and anti-inflammatory pathways.

## Acknowledgments

The authors are indebted to Binhai County People’s Hospital, which furnished a clinical medical research platform. This work was supported by Scientifc Research Project of the Yancheng Municipal Health Commission, Jiangsu Province, China (Grant Nos. YK2023109 and YK2024066) and Nantong University Special Research Fund for Clinical Medicine (Grant No. 2025JY022).

## Author contributions

**Data curation:** Chengchen Liu, Nan Gao, Xun Yan, Ming Zhang, Ye Hong, Bo Hou, Kangkang Ji, Feng Lu.

**Formal analysis:** Chengchen Liu, Nan Gao, Xun Yan, Ming Zhang, Ye Hong, Bo Hou, Kangkang Ji, Feng Lu.

**Investigation:** Chengchen Liu, Nan Gao, Xun Yan, Kangkang Ji, Feng Lu.

**Methodology:** Chengchen Liu, Nan Gao, Xun Yan, Kangkang Ji, Feng Lu.

**Project administration:** Nan Gao, Xun Yan, Kangkang Ji, Feng Lu.

**Resources:** Nan Gao, Xun Yan, Kangkang Ji, Feng Lu.

**Software:** Nan Gao, Xun Yan, Kangkang Ji, Feng Lu.

**Validation:** Nan Gao, Xun Yan, Kangkang Ji, Feng Lu.

**Writing – original draft:** Nan Gao, Xun Yan, Kangkang Ji.

**Conceptualization:** Xun Yan, Kangkang Ji, Feng Lu.

**Supervision:** Xun Yan, Kangkang Ji, Feng Lu.

**Visualization:** Xun Yan, Kangkang Ji, Feng Lu.

**Writing – review & editing:** Kangkang Ji, Feng Lu.


