# Supplementary material for: Dietary vitamin B6 intake and gallstone prevalence in US adults: A cross-sectional analysis of NHANES 2017–2023
Source: Medicine (Baltimore). 2026 Aug 14;105(33):e50208. doi: 10.1097/MD.0000000000050208 (PMC13480687; doi:10.1097/MD.0000000000050208)
Supplement: Supplementary file 1 [file medi-105-e50208-s001.docx]

**Table S1.** Subgroup and interaction analysis.

| **Subgroup** |  | OR 95CI% | P for interaction |
| --- | --- | --- | --- |
| **All patients** | | 0.91 (0.83, 0.99) |  |
| **Age group** | 20-39 | **0.75 (0.56, 1.00)** | 0.077 |
|  | 40-59 | 0.90 (0.77, 1.05) |  |
|  | 60+ | 0.98 (0.89,1.09) |  |
| **Race** | Hispanic | 0.95 (0.75, 1.19) | 0.899 |
|  | Non-Hispanic White | **0.89 (0.80, 0.99)** |  |
|  | Non-Hispanic Black | 0.87 (0.66, 1.16) |  |
|  | Other | 1.02 (0.84, 1.24) |  |
| **Education** | < HS | **0.66 (0.47, 0.92)** | 0.999 |
|  | HS diploma | 0.95 (0.85, 1.05) |  |
|  | College or above | 0.88 (0.78, 1.00) |  |
| **PIR** | <1 | 0.91 (0.80, 1.03) | 0.622 |
|  | 1 to 4 | 0.94 (0.84, 1.05) |  |
|  | >4 | **0.77 (0.59, 1.00)** |  |
| **Drinker** | Yes | **0.90 (0.82, 0.99)** | 0.851 |
|  | No | 0.93 (0.69, 1.25) |  |
| **Physical activity** | Sedentary | 0.93 (0.80, 1.08) | 0.469 |
|  | Moderate | 0.97 (0.88, 1.05) |  |
|  | Vigorous | **0.69 (0.55, 0.85)** |  |
| **Diabetes** | Yes | **0.77 (0.62, 0.95)** | 0.332 |
|  | No | 0.93 (0.85, 1.02) |  |
| **Hypertension** | Yes | **0.82 (0.70, 0.97)** | 0.720 |
|  | No | 0.95 (0.87, 1.04) |  |

Note: Subgroup analysis corresponds to a fully adjusted model, adjusted for age, gender, race, education, PIR, marital status, smoking, drinking, BMI, physical activity, diabetes, hypertension, total energy intake, and water intake.
